# Changes in Muscle Mass and Strength in Adolescents Following High-Intensity Functional Training with Bodyweight Resistance Exercises in Physical Education Lessons

**DOI:** 10.3390/jcm13123400

**Published:** 2024-06-11

**Authors:** Dawid Koźlenia, Marek Popowczak, Rafał Szafraniec, Cristian Alvarez, Jarosław Domaradzki

**Affiliations:** 1 Faculty of Physical Education and Sport, Wroclaw University of Health and Sport Sciences, 51-612 Wrocław, Poland; dawid.kozlenia@awf.wroc.pl (D.K.); marek.popowczak@awf.wroc.pl (M.P.); rafal.szafraniec@awf.wroc.pl (R.S.); 2 Exercise and Rehabilitation Sciences Institute, School of Physical Therapy, Faculty of Rehabilitation Sciences, Universidad Andres Bello, Santiago 7591538, Chile; cristian.alvarez@unab.cl

**Keywords:** adolescent, physical education and training, physical fitness, physical functional performance, muscle

## Abstract

**(1) Background**: The growing prevalence of obesity, diabetes, hypertension, and declining physical fitness among children and adolescents due to sedentary lifestyles has increased attention toward preventive intervention to tackle this issue. This study investigated the age-related effects of high-intensity functional training (HIFT), based on bodyweight resistance exercises conducted during physical education lessons, on muscle mass and strength improvement. **(2) Methods**: Adolescent males (n = 116) were allocated to four HIFT experimental groups (EGs) and four standard physical education program control groups (CGs) according to age (15, 16, 17, and 18 years [y]). The changes in muscle mass (absolute and relative to height [SMI]), hand–grip strength (HGS), sit-ups (SUs), and standing broad jump (SBJ) were analyzed using two-way analysis of variance (ANOVA) with Bonferroni tests. **(3) Results**: HIFT significantly increased muscle mass and scores in all strength tests (*p* < 0.01), while chronological age was significant for HGS (*p* < 0.01). Interactions between HIFT and chronological age categories were observed for HGS (*p* = 0.01) and SBJ (*p* < 0.03). Detailed post hoc tests revealed improvement in muscle mass across all chronological age categories for both approaches (*p* < 0.05). The 18y-EG group improved HGS over their control peers (*p* < 0.01), the EG groups significantly improved their SU results (*p* < 0.01), and SBJ improved in the 15y-EG and 18y-EG groups compared to their control (*p* < 0.01). **(4) Conclusions**: This research highlights the effectiveness of a school-based HIFT program in promoting muscle mass gains and enhancing muscle strength among adolescents. The findings offer valuable insights for implementing bodyweight exercises during physical education classes.

## 1. Introduction

The growing prevalence of obesity, diabetes, and hypertension and a decrease in physical fitness (PF) associated with sedentary lifestyles among children and adolescents have fueled a heightened interest in preventive actions to address this global problem [1]. Overweight and obesity during childhood and adolescence have multiple repercussions and contribute to significant health issues that continue in adult life. There is a tendency to remain overweight or obese from childhood to adulthood [2], which is associated with an increased risk of type-2 diabetes, hypertension, cardiovascular and metabolic disorders, and may ultimately result in early mortality [3,4].

Incorporating resistance training (RT) into initiatives aimed at improving the physical fitness of young people has proven effective [5]. Participating in RT once or twice weekly has been reported to reduce body fat, enhance PF, and promote a healthier lifestyle among adolescents [6]. Physical activity is the most effective way to improve PF, body composition, and metabolic control [7]. Therefore, the role of physical education (PE) lessons, which are natural settings for introducing physical activity to youth, is paramount [8]. Given the alarming prevalence of obesity, diabetes, and hypertension, and the need to work with health systems to prevent these diseases and promote health education among students, one of the important challenges facing education systems is the optimal use of 45 min PE lessons to achieve more health benefits for school-age children [9]. 

Recently, there has been a lot of interest in introducing intermittent/interval exercises into PE lessons, in which short-term intense physical activity is interspersed with periods of recovery, just as it usually happens when children play [10]. Also, some resistance exercise modalities introduced into PE have proven effective in muscular fitness development [11]. These programs also contribute to improvements in self-perceived physical abilities. Thus, considering the brief duration of PE lessons, including these exercises in a single lesson of the standard curriculum could promote positive health-related physiological adaptations and collaborate to decrease obesity and other cardiometabolic conditions [12]. For example, it is widely known that several high-intensity interval training (HIIT) protocols, defined as short intervals of exercise at high intensity interspersed by recovery periods, are a powerful strategy to decrease adiposity, insulin resistance, and blood pressure [13]. However, HIIT strategies usually require exercise equipment that is not accessible at all schools. As such, the effects of a relatively recent workout approach termed high-intensity functional training (HIFT), a training style that integrates multiple functional movements performed intermittently at a high intensity (relative to individual capabilities) aimed at enhancing health-related fitness (body composition) and PF performance (muscle power and strength), require more understanding. HIFT emphasizes whole-body movements, incorporates aerobic and resistance exercises [14], and can be adapted to stimulate muscle growth and cardiorespiratory fitness [15,16,17]. Furthermore, incorporating RT programs was shown to increase PF and body composition effectively in school-age individuals [18].

There is little information regarding short HIFT regimes developed into circuit protocols and applied to school PE curricula and after-school fitness initiatives [19]. It seems that HIFT can improve physical and mental health, including muscle strength and self-esteem, and offers a valuable update for high school PE programs despite some challenges, such as engagement. Overcoming these obstacles can make HIFT an impactful part of PE [20]. The primary HIFT concept is work capacity over time, including diverse exercise modalities such as various bodyweight movements (squats, push-ups, etc.) [16,17]. The intermittent intensive exercise intervention may promote broad positive adaptation and effectively promote health in school settings [21]. Bodyweight modalities are the most suitable and frequently applied RT exercises in school environments [22]. The multiple exercises included in HIFT regimes also provide positive adaptations in cardiorespiratory fitness, a reduction in body fat, and an improvement in bone mineral content [15,23]. However, more attention should be paid to the implementation of HIFT in schools [20]. 

Chronological age is determined by the date of birth. Although biological age is a better indicator of maturity and is widely used in youth sports, its usefulness in school settings is limited [24,25]. Therefore, using chronological age is a more practical approach in schools, as students are grouped into classes based on their birth year. In this context, when implementing a training intervention in natural school settings, chronological age must be considered as a factor that could potentially influence the results of the intervention [26]. 

The effectiveness of developing muscle mass and strength among adolescents within natural PE environments using the HIFT concept remains unclear. Additionally, it is unknown whether age-related differences in effect occur among secondary school adolescents, which could indicate the need for different training interventions [20]. It could be suspected that older ones could be more responsive to resistance exercises in terms of muscle mass and strength development, but there is the question of younger adolescents also benefitting from this type of intervention. Elevated muscle mass correlates with improved metabolic status, while fitness levels expressed as muscle strength are indicative of overall health [1,5,23]. Developing physical fitness in youth allows for maintaining it in the subsequent years of life. This indicates the need to examine the potential for inducing positive adaptations in muscle mass and strength [6]. Emphasizing RT in youth fitness programs is crucial for developing essential muscular strength and motor skills and fostering confidence and competence for sustained physical activity throughout life [22,27]. Therefore, this study aimed to investigate the age-related effects of HIFT on (1) changes in muscle mass and (2) muscle strength. We hypothesized that all age groups would achieve improvements in muscle mass and strength, but older individuals would benefit more. Our results will clarify whether HIFT bodyweight exercises can increase muscle mass gains and strength.

## 2. Materials and Methods

### 2.1. Ethics

The project received approval from the Ethics Committee of the Wroclaw University of Health and Sport Sciences (No. 33/2018 on the date 31 October 2018). Comprehensive information regarding the study’s design, including potential risks and benefits, was provided to all participants and their legal guardians before receiving their signed informed consent to participate. Subsequently, all legal guardians were requested to sign a consent document before the commencement of testing. All participants were allowed to withdraw from the project without providing any reasons.

### 2.2. Participants

Data from 116 individuals were analyzed. In this term, for a general linear multidimensional analysis of variance (MANOVA) involving eight groups, two repeated measures, and their interaction term, we set an effect size (ES) of 0.36, a significance level of 0.05, and a power of 0.80 was estimated. Participants were recruited from one school. They were of similar sociocultural backgrounds and resided in the same large city with approximately 650,000 citizens. A simple, non-returnable group randomization was performed utilizing the tool available on www.randomization.com. The study was performed in natural school settings. To maintain the integrity of the educational process with minimal disruption, we could not select individual adolescents but used entire classes. Each class was coded according to its level (e.g., 1A, 2B, etc.). Two classes were randomly selected from each level (1–4). The participants were divided into eight distinct classes, with two classes per level. Subsequently, one class from each level was randomly assigned to either the experimental group or the control group. 

The study sample comprised 116 adolescent boys aged 15–18, divided into experimental groups (EGs) and control groups (CGs), recruited from a predetermined urban comprehensive secondary school. The exclusion criteria included participation in organized after-school physical activity (planned and supervised physical activities that take place outside of school hours conducted by sports clubs or other institutions under the supervision of instructors or coaches), medical contraindications for physical activity, cardiovascular/respiratory diseases, and the discontinuation of PE class participation due to school or class changes. However, none were excluded due to the established criteria. The adolescents were classified into four categories based on chronological age, including 15 years (15y), 16y, 17y, and 18y. Of those who met the conditions, 30 individuals from every age category were randomly stratified into EG or CG groups for each age group. This approach was driven by the need to maintain natural school conditions and avoid significant interference with the organization of PE lessons. Detailed participant data are provided in Table 1 in the results section. Participants were asked to maintain their routine levels of physical activity and behaviors [28]. After starting the project, the results of four participants (16y-EG n = 1 and 18y-CG n = 3) were excluded due to failure to complete all tests. None of the participants withdrew from the program due to fatigue, health issues, or a lack of interest.

### 2.3. Intervention

In all experimental and control groups, a single PE lesson duration time was 45 min. The lesson started with a standardized 10 min warm-up, included 5 min of slow jogging and 5 min of static and dynamic stretching. The lesson was finished with 5 min of flexibility and relaxation exercises. In the main part of PE in the control group, the standard curriculum was realized where individuals enhanced their physical literacy and developed skills in various sports, such as volleyball, basketball, football, gymnastics, athletics, dancing, table tennis, and Nordic walking. In the EG, after the standard warm-up and before the standard content of physical education lessons, the intervention program was introduced.

**Figure 1:**
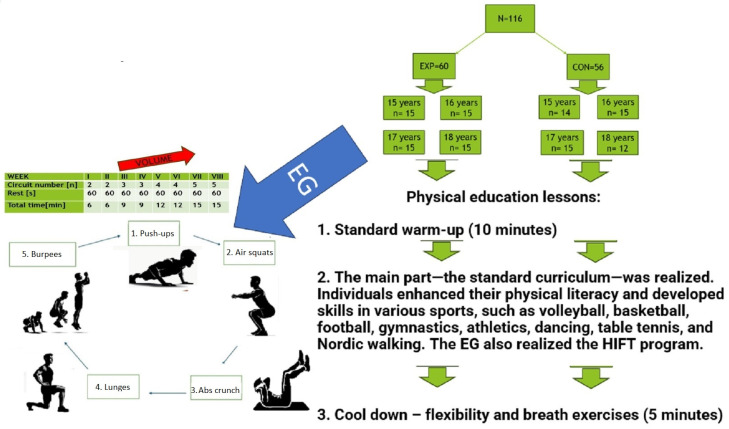
Study intervention design.

HIFT was based on performing a circuit consisting of squats, abs crunches, push-ups, lunges, and burpees. Participants performed each exercise for 20 s without breaks between them. Participants were instructed to perform as many reps as possible while maintaining proper technique. The teacher supervised and corrected participants when needed. A 60 s rest was allowed after the entire circuit. The duration of the HIFT session was 6 min in the first two weeks and 14 in the last two weeks. During the first two weeks, participants performed two circuits, and then one circuit was added every two weeks, meaning participants performed five circuits in the final week. The volume progression patterns are presented in Table 1. The program was progressively overloaded based on volume (Figure 1). Individuals undertook the standard PE program in the rest of the lessons and the third lesson of the week. In every exercise session, the Rating of Perceived Exertion (RPE) scale was utilized to assess effort level, fatigue, and training load, aiming for a score of 7–8 on a scale that is a valid method of intensity monitoring among youth in school settings [29]. The details of the realized intervention are also presented in Figure 1.

### 2.4. Physical Assessment

#### 2.4.1. Body Morphology: Muscle Mass Assessment

Anthropometric measurements used anthropometers (GPM Anthropological Instruments) to record two body height measurements with an accuracy of 0.1 cm. Body height was assessed following the protocol outlined by the International Society for the Advancement of Kinanthropometry (ISAK) [30]. Trained staff conducted the measurements, with their performance quality assessed against the standards in the ISAK Manual. 

Body weight and skeletal muscle mass were determined using the bioelectric impedance method by employing the InBody230 body composition device (InBody Co. Ltd., Cerritos, CA, USA). The device is renowned for its high reliability in males and females, as demonstrated by an elevated intraclass correlation coefficient (ICC) (≥0.99) and a low standard error of measurement [31]. InBody is characterized by high reliability and repeatability; therefore, it could be successfully utilized in repeated measures [32]. The above data were used to calculate the following indices:

Body Mass Index (BMI) = body mass [kg]/body height [m^2^]

Skeletal Muscle Mass Index (SMI) = body skeletal muscle mass [kg]/body height [m^2^]

#### 2.4.2. Hand–Grip Strength (HGS)

Hand–grip strength (HGS) was evaluated using a hydraulic hand dynamometer (Baseline, FEI, New York, NY, USA). Each participant’s device was adjusted individually, aligning its spine with their thumb. Participants gripped the handle firmly for 3–5 s with a neutral wrist, using chalk if preferred. Any pumping of the dynamometer was considered unsuccessful and prompted a retest to prevent artificially elevated readings. The best score from two trials was recorded for each hand.

#### 2.4.3. Sit-Ups (SU)

For the sit-ups (SU) test, participants performed sit-ups for 30 s with hands placed at their head’s sides, knees bent at a 90-degree angle, and feet secured. A successful sit-up involved touching the knees with elbows and returning the shoulders to the ground. The total number of accurate sit-ups within the timeframe was documented, with evaluators announcing the remaining time at 10-, 20-, and 30-s intervals. Verbal encouragement was provided, and each participant underwent the test once.

#### 2.4.4. Standing Broad Jump (SBJ)

Regarding the Standing Broad Jump (SBJ), participants faced a designated line and executed a jump using upper limb swinging motion, landing on both legs. The jump length was measured from the line’s reflected edge on the heels, with two attempts allowed, and the better result considered. Measurements were precise to 0.5 cm.

All measurements were conducted on the same day between 8:00 a.m. and 1:00 p.m. in sports halls, ensuring identical conditions for each group. Participants wore T-shirts, shorts, and sports footwear, with anthropometric measurements taken without sneakers. Anthropometric measurements preceded muscle strength assessments, following H-RF guidelines and ensuring the scientific rationale behind test selection, and their reliability in young individuals was confirmed beforehand [33].

### 2.5. Statistics

The normality of the distribution of the analyzed variables was evaluated using the Shapiro–Wilk test. None of the variables violate the assumption of a normal distribution. Therefore, parametric methods were applied. Means and standard deviations were computed, and confidence intervals (95%CI) were provided to provide descriptive statistics of the analyzed data. The variance homogeneity was confirmed, and the two-way analysis of variance (ANOVA) for baseline measures was conducted, and the post hoc Bonferroni test was introduced to check whether the differences in one age category that expressed school class (15y; 16y; 17y; 18y) appeared between experimental (EG) and control (CG) groups. Then, to confirm whether the introduced intervention brought any significant effects expressed as changes (post-intervention–baseline difference [Δ]), the multiple analysis of variance (MANOVA) was used to assess the global effects of training in repeated measurements. Then, the next two-way ANOVA for changes (Δ) was applied to analyze the Δ magnitude considering the condition (EG–CG) and calendar age category. The partial eta-square (η^2^_p_) was calculated and interpreted as 2p = 0.01, a small effect; 2p = 0.06, a medium effect; and η2p = 0.14 indicates a large effect. When a significant F-ratio was revealed, a post hoc Bonferroni test was performed to deepen the analysis of the observed changes in the study groups. The significance level assumed in all statistical tests was *α* = 0.05. Statistica v13.0 (Statsoft Polska, Cracow, Poland) was used for statistical analysis.

## 3. Results

Table 1 presents descriptive statistics for analyzed morphological and motor parameters considering group adherence.

**Table 1:** Descriptive statistics. Data are shown as mean and ±SD. Groups are described based on condition (EG or CG) and age (15, 16, 17, or 18).

Outcomes	15y-EG, n = 15	16y-EG, n = 14	17y-EG, n = 15	18 y-EG, n = 15	15 y-CG, n = 15	16 y-CG, n = 15	17 y-CG, n = 15	18 y-CG, n = 12
Statistics	Mean ± SD (95%CI)							
BH [cm]	173.4 ± 6.8	179.6 ± 7.8	179.6 ± 5.2	179.2 ± 6.2	172.5 ± 6.2	178.1 ± 4.8	180.4 ± 3	180.5 ± 5
	(169.6–177.1)	(175.2–183.9)	(176.7–182.5)	(175.8–182.7)	(168.9–176.1)	(175.4–180.8)	(178.8–182.1)	(177.3–183.6)
Δ BH [cm]	0.3 ± 0.4	0.4 ± 0.4	0.5 ± 0.5	0.4 ± 0.5	0.2 ± 0.2	0.2 ± 0.2	0.2 ± 0.3	0.3 ± 0.3
	(0.1–0.5)	(0.2–0.6)	(0.2–0.8)	(0.1–0.7)	(0.1–0.3)	(0.1–0.3)	(0.1–0.4)	(0.1–0.5)
BW [kg]	73.5 ± 13.5	72.2 ± 9.9	70.8 ± 6.6	73.3 ± 12.5	65.7 ± 9	74.3 ± 10.4	68.3 ± 5.6	65.9 ± 6.9
	(66–80.9)	(66.8–77.7)	(67.1–74.4)	(66.4–80.1)	(60.5–70.9)	(68.6–80)	(65.2–71.4)	(61.5–70.3)
Δ BW [kg]	0.0 ± 1.1	−0.2 ± 1.9	−0.2 ± 1.6	0.2 ± 1.2	0.5 ± 0.5	−0.4 ± 1.3	0.1 ± 0.6	1.1 ± 1.4
	(−0.6–0.6)	(−1.3–0.9)	(−1.1–0.7)	(−0.4–0.8)	(0.2–0.7)	(−1.1–0.3)	(−0.2–0.4)	(0.2–2)
BMI [kg/m^2^]	24.3 ± 3.5	22.4 ± 2.7	21.9 ± 1.8	22.8 ± 3.7	22.1 ± 3	23.5 ± 3.5	21 ± 1.6	20.2 ± 1.7
	(22.4–26.3)	(20.9–23.9)	(21–22.9)	(20.7–24.8)	(20.4–23.8)	(21.5–25.4)	(20.1–21.8)	(19.1–21.3)
Δ BMI [kg/m^2^]	−0.3 ± 0.8	−0.1 ± 0.6	−0.2 ± 0.5	−0.1 ± 0.4	0.1 ± 0.2	−0.2 ± 0.4	0 ± 0.2	0.3 ± 0.4
	(−0.7–0.2)	(−0.5–0.2)	(−0.4–0.1)	(−0.3–0.1)	(0–0.2)	(−0.4–0.0)	(−0.1–0.1)	(0–0.5)
SMM [kg]	24.3 ± 3.6	24.8 ± 2.7	25.1 ± 2	25.7 ± 2.8	24.2 ± 3.9	27 ± 2.8	26.2 ± 2.1	26.4 ± 2.7
	(22.3–26.3)	(23.3–26.3)	(24–26.2)	(24.1–27.3)	(22–26.5)	(25.5–28.6)	(25–27.3)	(24.8–28.1)
Δ SMM [kg]	0.6 ± 0.8	1.1 ± 0.7	1.0 ± 0.6	0.8.0 ± 0.6	−0.5 ± 0.4	−0.6 ± 0.6	−0.5 ± 0.5	−0.6 ± 0.6
	(0.2–1.1)	(0.8–1.5)	(0.7–1.3)	(0.5–1.1)	(−0.7–−0.3)	(−0.9–−0.3)	(−0.8–−0.2)	(−1–−0.2)
SMI [kg/m^2^]	8.1 ± 0.8	7.7 ± 0.6	7.8 ± 0.6	8 ± 0.6	8.1 ± 1	8.5 ± 1.1	8.0 ± 0.6	8.1 ± 0.7
	(7.6–8.5)	(7.3–8)	(7.5–8.1)	(7.7–8.3)	(7.5–8.7)	(8–9.1)	(7.7–8.4)	(7.6–8.6)
Δ SMI [kg/m^2^]	0.2 ± 0.3	0.3 ± 0.2	0.3 ± 0.2	0.2 ± 0.2	−0.2 ± 0.1	−0.2 ± 0.2	−0.2 ± 0.2	−0.2 ± 0.2
	(0–0.3)	(0.2–0.4)	(0.2–0.4)	(0.1–0.3)	(−0.3–−0.1)	(−0.3–−0.1)	(−0.3–−0.1)	(−0.4–−0.1)
HGS [kg]	43.2 ± 10.9	48.7 ± 8.4	46.7 ± 8.2	54.3 ± 9.4	36.2 ± 7.8	45.6 ± 7.9	48 ± 5.9	51.9 ± 7
	(37.2–49.2)	(44–53.3)	(42.1–51.2)	(49.1–59.5)	(31.7–40.7)	(41.3–49.9)	(44.7–51.3)	(47.5–56.3)
Δ HGS [kg]	−0.5 ± 2.1	0.7 ± 2.4	2.3 ± 2.7	2.5 ± 2.6	−0.9 ± 3.7	0.9 ± 2.8	1.6 ± 2.6	−1.9 ± 2.1
	(−1.7–0.6)	(−0.6–2.1)	(0.8–3.8)	(1.1–4)	(−3.1–1.2)	(−0.6–2.5)	(0.2–3.0)	(−3.3–−0.6)
SU [reps/30 s]	22.3 ± 5.1	24.3 ± 4.2	25.3 ± 5.2	23.7 ± 5.0	23.6 ± 3.4	25.4 ± 4.4	25.3 ± 4.9	22.6 ± 4.8
	(19.5–25.1)	(21.9–26.6)	(22.5–28.2)	(21–26.5)	(21.6–25.5)	(23–27.8)	(22.6–28)	(19.5–25.6)
Δ SU [reps/30 s]	1.5 ± 2.5	3.3 ± 5.1	4.1 ± 3.9	3.6 ± 4.3	−0.1 ± 1.6	0.3 ± 2.3	0.1 ± 4.8	1 ± 2.1
	(0.1–2.8)	(0.4–6.1)	(1.9–6.2)	(1.2–6.0)	(−1.0–0.8)	(−1.0–1.6)	(−2.5–2.8)	(−0.4–2.4)
SBJ [cm]	186.7 ± 28.4	197.5 ± 25.7	201.9 ± 30.7	205.9 ± 27.5	188.3 ± 25.3	198.3 ± 35.4	192.5 ± 18.6	202.3 ± 23.3
	(171–202.4)	(183.3–211.8)	(184.9–218.9)	(190.7–221.2)	(173.7–202.9)	(178.7–217.9)	(182.2–202.9)	(187.5–217.2)
Δ SBJ [cm]	13.6 ± 14.4	5.8 ± 10.8	8.3 ± 12.9	11.3 ± 11.5	−3.9 ± 8.4	2.8 ± 5.9	5.3 ± 5.6	3.0 ± 9.4
	(5.6–21.6)	(−0.2–11.8)	(1.2–15.5)	(4.9–17.6)	(−8.8–0.9)	(−0.5–6.1)	(2.2–8.4)	(−3.0–9.0)

Abbreviations: EG—Experimental Group; CG—Control Group; Δ—Change (post-pre difference); BH—Body Height; BW—Body Weight; BMI—Body Mass Index; SMM—Skeletal Muscle Mass; SMI—Skeletal Muscle Mass Index; HGS—Hand Grip Strength; SU—Sit-Ups; SBJ—Standing Broad Jump; SD—Standard Deviation.

The two-way ANOVA for baseline data differences assessment was applied to check whether baseline differences within age categories occur. The age category and conditions state a statistically significant effect (*p* > 0.05). However, detailed comparisons with the Bonferroni post hoc test did not reveal any significant differences in all analyzed parameters within the age categories. The analyzed experimental and control groups in one age category did not differ in any of the examined parameters (*p* > 0.05).

In the next step of the analysis, MANOVA was performed, which showed multidimensional results (Δ magnitude) of comparisons between all groups. Calculated statistics confirmed a good explanation of the variable model differences between groups (Wilks’ Λ = 0.76, (η^2^_p_ = 0.09, *p* = 0.01). The next step was to perform a two-way ANOVA. Table 2 presents results for the two-way ANOVA for the main effects of condition (EG and CG) and calendar age category (15, 16, 17, and 18) and their interactions. The effect of the condition was significant for all analyzed parameters (*p* < 0.01). The value of 2p indicated a large effect for all analyzed parameters, besides HGS, where medium effects were noted. Age category was significant for hand–grip changes (*p* < 0.01) with medium effect. Both factors interacted for HGS (*p* < 0.01) and SBJ (*p* < 0.03) with medium effects in both cases.

**Figure 2:**
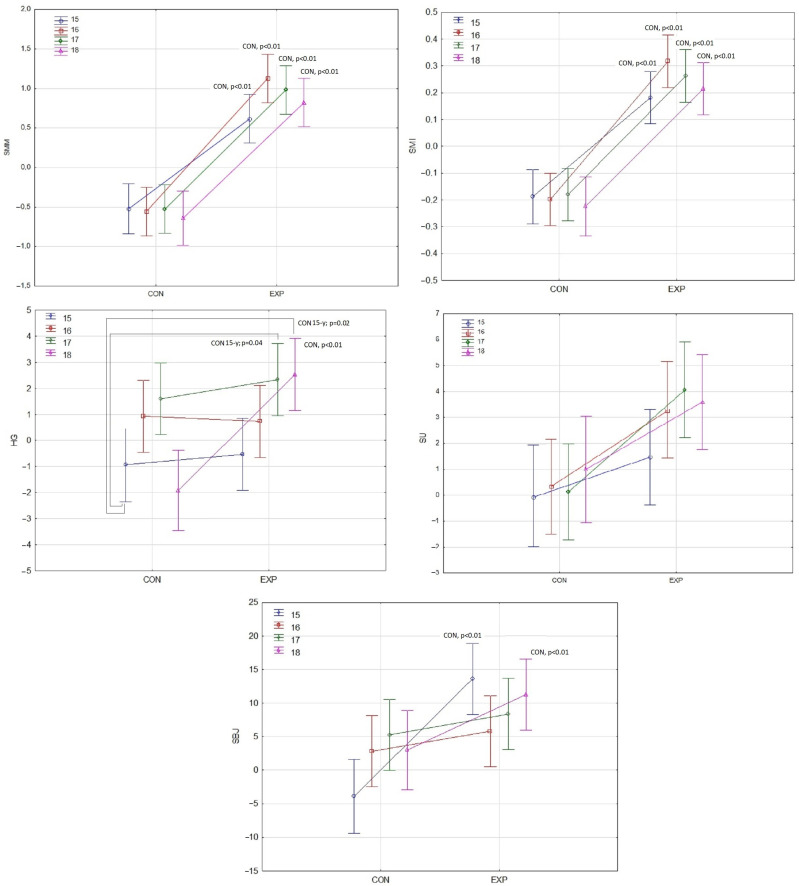
Changes in muscle mass and motor tests. Points represent means, and bars indicate 95% confidence intervals. Abbreviations: CON—control group; EXP—experimental group; Δ—Change (post-pre difference); SMM—Skeletal Muscle Mass; SMI—Skeletal Muscle Mass Index; HGS—Hand–Grip Strength; SU—Sit-Ups; SBJ—Standing Broad Jump. *p* < 0.05 statistically significant differences. CON—statistically significant higher results than the related control group. AGE-y—statistically significant higher results than the indicated (y) age group.

The post hoc Bonferroni test revealed significant differences between all experimental and control groups (*p* < 0.01) in the case of the muscle mass absolute and relative to the height. For changes in HGS, the differences were not as prevalent as muscle mass changes. The 17y-EG group had more gains than the 15y-CG (*p* = 0.04), while the 18y-EG improved their hand grip more than the 15y-CG (*p* = 0.02) and 18y-CG (*p* < 0.01). For trunk muscle strength, expressed as the number of repetitions of sit-ups performed during 30 s, only a significant effect was observed for the condition (experimental control) effect. The SBJ improved in the 15y-EG and 18y-EG groups compared to the 15y-CG and 18y-CG, respectively (*p* < 0.01). The described changes are presented in Figure 2.

## 4. Discussion

This study aimed to investigate the age-related effects of HIFT using bodyweight RT conducted during PE lessons on muscle mass and strength improvement, considering potential differences due to calendar age. Our results showed positive changes in muscle mass gains across all experimental age groups after the intervention. Also, strength improvement was observed in HGS and SBJ, with the most visible gains in the 18y-EG. As such, our results demonstrate that the proposed HIFT protocol could be implemented in PE lessons for favorable body morphology and physical performance adaptations in secondary school children. The observed changes are associated with positive adaptations in body morphology and PF that could prevent metabolic issues. Our work presents some unique observations. Although many authors have addressed the topic of various forms of short, intensive programs implemented within physical education, there is still a lack of studies that explore this topic in the context of HIFT. Moreover, the issue of improving muscle mass and strength among adolescents during PE lessons is also not explored.

Strength is a base for PF and enables individuals to handle external loads crucial for sports performance and daily activities [34]. Thus, prioritizing strength development is essential across all age groups. In youth, strength training supports physical growth and lays the groundwork for maintaining PF later in life. At the same time, in adults, it assists in meeting occupational demands and combating sarcopenia in older age [35].

HIFT programs contribute to beneficial muscle tissue adaptations and enhanced motor performance, or even mental state, which was observed as attention ability [36]. In other forms, intensity efforts may bring improvement in cognitive function [37]. Previous research confirmed that short, intensive efforts positively affect muscular strength and function in healthy adolescents, suggesting that such training interventions during PE lessons can be as effective as longer ones [38]. Lastly, it was shown that athletes may achieve many benefits through HIFT [39]. However, our results confirmed the ability to comprehensively implement this exercise in schools. The short-term intensive intervention improved endurance and musculoskeletal fitness (MSF) and reduced BMI and body fat percentage (BFP) [12] when implemented in PE lessons. Additionally, it positively affected muscle mass, strength, and cardiovascular parameters [40]. Our study highlighted the positive impact of a HIFT intervention on muscular development across various school-age groups. These results underscore strength improvement as a prominent effect of the various short-interval training programs implementing resistance efforts [41]. Positive adaptations were observed in a broad spectrum of PF, especially strength abilities, after the HIFT program was introduced among adolescents [42]. In line with our results, Engel et al. [43] also observed that HIFT sessions improved strength capacity in adolescence.

Studies by Agostinis-Sobrinho et al. [44] indicated that enhancing muscle strength reduces cardiometabolic risk in youth, notably decreasing adolescent blood pressure. A review by Abarzúa et al. [45] summarized the positive effects of interval training on musculoskeletal fitness in teenagers, suggesting it may be more effective than continuous training [46]. Moreover, interval training increases muscle mass in obese individuals, albeit requiring appropriate training loads [47]. Research by Cvetković et al. [28] demonstrated positive effects on muscle mass, similar to our findings and those of Teixeira et al. [48], where increased power was observed. Muscle mass is closely tied to a higher metabolic state, reflecting a more remarkable ability to exert effort during workouts [49], and insufficient effort may lead to a lack of positive adaptations. 

HGS correlates with muscle strength, suggesting that, in clinical practice, grip strength serves as a quick indicator of an individual’s overall muscle strength [50]. Increased grip strength is linked to sustained health and health enhancements over time in adolescents. Conversely, low grip strength could be a predictive marker for cardiometabolic risk, helping identify adolescents who stand to gain the most from lifestyle interventions to enhance muscular fitness [51]. 

Many studies have shown the advantages of HIFT programs, including enhanced aerobic capacity, bone health, muscle strength, and muscle mass increments [23,52,53,54]. HIFT also brings mental health wellness by raising brain-derived neurotrophic factor levels, potentially aiding memory and learning, which may bring educational benefits [17] and elevate life quality [55]. HIFT also promotes self-esteem in teens facing psychological challenges [56], highlighting its broader impact on adolescent health and academic success. 

Despite all age categories responding positively to HIFT, older boys gained more in terms of strength. Alvarez et al. [57] showed that the effects after the exercise program could be independent of maturation. Generally, calendar age or maturation is essential to a youth’s morphology and functional state [33], though the influence of biological age on the effect of short-term intensive intervention remains unclear. Nonetheless, more mature individuals are more morphologically developed and present higher PF, which is associated with muscle mass and nervous system development [58]. However, data must still be included that consider the maturation status of the effects of various short, intensive interventions on school-age populations [59]. Despite biological maturation being a more valuable factor than simple calendar age, natural school settings forced the consideration of this factor. At school, children and adolescents are assigned to classes based on their date of birth; therefore, in this type of intervention, calendar, and age must be considered as factors influencing the research. Of course, maturation is strongly associated with calendar age, but there are reports about biological age as a valuable factor in youth sports in school settings. Its usefulness is limited. However, the division based on chronological age is a generally accepted approach in school settings. [60]. An additional factor that must also be considered in HIFT effects analysis is the motivation to undertake intensive exercise. Some individuals may avoid fatigue, meaning the magnitude of the stimulus is too low for positive adaptations [61,62]. As such, further studies are warranted.

Acknowledging study limitations, we recognize that the participation of only one secondary school limits the generalizability of our findings. A wider age range would provide more specific results. Additionally, we needed more control over nutrition, did not monitor average daily physical activity, and did not continuously record heart rate during subsequent lessons. We did not assess biochemical parameter status (e.g., testosterone), which could provide deeper insight into our study. Also, we did not consider the biological age of the participants, and only boys participated in the study. We did not assess the participants’ well-being. Another limitation is that an improvement in sit-ups was to be expected in the EC when exercise in the form of crunch-ups, which has a very similar movement pattern, was explicitly trained. Indicated limitations should be addressed in a future study. However, strengths include the study’s natural school setting, demonstrating the feasibility of implementing HIFT in PE programs, and the heterogeneous sample of individuals at similar educational levels and ages. Individuals’ ability to perform specific exercises is crucial to tailoring optimal exercise interventions for health development. Future studies should address these limitations to gain deeper insights into individual responses to HIFT interventions based on MF profiles. Additionally, future studies should include girls and a more comprehensive age range to provide more generalizable results. Also, it would be interesting to perform other forms of training programs during PE lessons to assess the changes in muscle mass and strength [63]. 

## 5. Conclusions

The significant contributions of this research include providing evidence for the effectiveness of a school-based HIFT intervention program on muscle mass gains and strength increase across all experimental age groups. The most superior enhancement was gained for those aged 18, who had more improvements than their peers, although maturity is associated with a higher responsiveness to this type of intervention. As such, there is a need to clarify a more optimal regime for younger boys. The observed changes indicate favorable adaptations associated with a decreased risk of obesity and other metabolic health issues. The findings from this study could assist teachers in evaluating both the potential and indirect effects on muscle performance following the implementation of a bodyweight intervention grounded in the HIFT concept during physical education classes. These findings allow teachers to provide a more targeted approach for treating adolescents to promote muscle mass gains in parallel with the effects on muscle strength and power. Future studies need to optimize the intervention to achieve more gains in strength in younger groups.

## Figures and Tables

**Table 2:** Two-way ANOVA. Main effects and interaction results.

Statistics	Δ SMM [kg]			Δ SMI [kg/m^2^]			Δ HGS [kg]			Δ SU [Reps/30 s]			Δ SBJ [cm]		
	F	*p*	η^2^_p_	F	*p*	η^2^_p_	F	*p*	η^2^_p_	F	*p*	η^2^_p_	F	*p*	η^2^_p_
Condition	168.38	0.01	0.61	154.13	<0.01	0.59	7.2	0.01	0.06	16.9	<0.01	0.14	17.1	<0.01	0.14
Age category	1.01	0.39	0.03	0.82	0.48	0.02	5.12	<0.01	0.12	1.13	0.34	0.03	0.53	0.66	0.01
Condition—Age category	1.07	0.37	0.03	0.72	0.54	0.02	4.2	0.01	0.10	0.56	0.65	0.02	3.19	0.03	0.08

Abbreviations: Δ—Change (post-pre difference); SMM—Skeletal Muscle Mass; SMI—Skeletal Muscle Mass Index; HGS—Hand Grip Strength; SU—Sit-Ups; SBJ—Standing Broad Jump.

## Data Availability

Data for the current study will be available upon reasonable request from the corresponding author.
